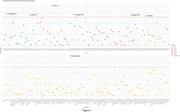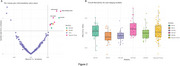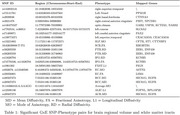# Genome‐Wide by Environment Interaction Studies of PM_2.5_ and Alzheheimer's Genetic Risk Across Brain Imaging Phenotypes in UK Biobank

**DOI:** 10.1002/alz70856_097952

**Published:** 2025-12-24

**Authors:** Zehui Sun, Tengfei Li, Samir Kelada, Jason L Stein, Hongtu Zhu

**Affiliations:** ^1^ University of North Carolina at Chapel Hill, Chapel Hill, NC, USA

## Abstract

**Background:**

Genome‐wide association studies have provided insights into single nucleotide polymorphisms (SNPs) which have been validated to be associated with risk for Alzheimer's disease (AD). However, their precise functions remain unknown. Recent research highlighted the impact of air pollutants such as PM_2.5_, on pathological progression of AD. In this context, we performed gene‐environment (GxE) study of PM_2.5_ and brain imaging‐derived phenotypes (IDPs) in UK Biobank to explore the role of air pollutants in genetic risk of AD.

**Method:**

We obtained volumetrics data of 101 regions of interest (*N* = 47,258) and diffusion imaging metrics of 110 white matter tracts (*N* = 45,522) for unrelated white‐ancestry subjects from UK Biobank. All autosomal genotypes passed standard quality control. PM_2.5_ estimates (in μg/m^3^) for the year 2010 were modelled for each address using a Land Use Regression (LUR) model developed as part of the European Study of Cohorts for Air Pollution Effects (ESCAPE). We applied interaction tests (GEM v1.5.3) on each IDPs. Polygenic score (PGS) for AD was obtained from UK Biobank (Field ID 26206). The interaction effect and heritability of GxE were calculated and tested using PIGEON where Functional Mapping and Annotation online platform (FUMA v1.6.2) and Multi‐marker Analysis of GenoMic Annotation (MAGMA) were employed to do post‐GWAS analysis.

**Result:**

We identified six significant PGS‐by‐environment interactions between PM_2.5_ and AD polygenic score (*p < 0.05*) in both regional volumes and white matter tracts (Figure 1). Heritability of interaction between polygenic structure and PM_2.5_ was further calculated for each IDP. We observed ten significant heritability of interaction effects (*p < 0.05*) (Figure 2A) and also obtained overall mean of heritability for each imaging modality (Figure 2B). At the genome‐wide threshold of 5 × 10^−8^, we identified 23 lead SNPs interacting with PM_2.5_ which are associated with 11 white matter microstructure traits and 8 regional volumes alongside the mapped genes (Table 1).

**Conclusion:**

This study delves into the gene‐environment effect of PM_2.5_ in genetic architecture of Alzheimer's disease, identifying novel risk loci associated with GxE effects. Our findings illustrate a big picture of genetic architecture of different brain structures and functions interacting with common air pollutants like PM_2.5_.